# Simvastatin decreases the level of heparin-binding protein in patients with acute lung injury

**DOI:** 10.1186/1471-2466-13-47

**Published:** 2013-07-19

**Authors:** Daniel F McAuley, Cecilia M O’Kane, Thelma R Craig, Murali Shyamsundar, Heiko Herwald, Karim Dib

**Affiliations:** 1 Regional Intensive Care Unit, Royal Victoria Hospital, Belfast, Northern Ireland, UK; 2 Centre for Infection and Immunity, Queen’s University of Belfast, Belfast, United Kingdom; 3 Department of Clinical Sciences, Division of Infection Medicine, University of Lund, Lund, Sweden

**Keywords:** Acute lung injury, Simvastatin, Heparin-binding protein, Inflammation, Neutrophils

## Abstract

**Background:**

Heparin-binding protein is released by neutrophils during inflammation and disrupts the integrity of the alveolar and capillary endothelial barrier implicated in the development of acute lung injury and systemic organ failure. We sought to investigate whether oral administration of simvastatin to patients with acute lung injury reduces plasma heparin-binding protein levels and improves intensive care unit outcome.

**Methods:**

Blood samples were collected from patients with acute lung injury with 48 h of onset of acute lung injury (day 0), day 3, and day 7. Patients were given placebo or 80 mg simvastatin for up to 14 days. Plasma heparin-binding protein levels from patients with acute lung injury and healthy volunteers were measured by ELISA.

**Results:**

Levels of plasma heparin-binding protein were significantly higher in patients with acute lung injury than healthy volunteers on day 0 (p = 0.011). Simvastatin 80 mg administered enterally for 14 days reduced plasma level of heparin-binding protein in patients. Reduced heparin-binding protein was associated with improved intensive care unit survival.

**Conclusions:**

A reduction in heparin-binding protein with simvastatin is a potential mechanism by which the statin may modify outcome from acute lung injury.

**Trial registration:**

Current controlled trials: ISRCTN70127774

## Background

Acute lung injury (ALI), and its most severe form, the adult respiratory distress syndrome (ARDS) are inflammatory disorders which are commonly caused by the systemic release of cytokines and pro-inflammatory molecules [1]. In the United States, there are approximately 190,000 cases per year of ALI with an associated death of 74,500 per year [1]. ALI is characterized by alveolar epithelial and capillary endothelial barrier damage, resulting in exudation of protein-rich oedema fluid into the alveolar space, which causes functional impairment of the gas exchange [1]. Uncontrolled and excessive infiltration of inflammatory cells, in particular neutrophils and macrophages, is a hallmark of ALI. Neutrophils are the main contributor in the pathogenesis of ALI. Indeed, there is a correlation between the number of neutrophils in bronchoalveolar (BAL) fluid and the severity of lung injury [2] and persistence of neutrophils is associated with mortality [3]. Neutrophils contribute to lung tissue destruction in ALI by producing tissue damaging agents such as reactive oxygen species, and granule-derived proteases [1].

In ALI, extravasation of neutrophils into the alveolar space is associated with disruption of the endothelial barrier. The mechanisms by which neutrophils induce change in alveolar and capillary endothelial barrier permeability is not well known, but may involve, at least in part, heparin-binding protein (HBP).

HBP, also known as CAP37/azurocidin, is a 28 kDa protein belonging to the serprocidin subgroup of the chymotrypsin-like proteases [4]. HBP is contained in azurophilic granules and secretory vesicles of neutrophils [5]. HBP is released upon fusion of azurophilic granules and secretory vesicles with the plasma membrane. The basal release of HBP is augmented upon β2 integrin-dependent adhesion of neutrophils to the vascular endothelium during inflammation [4]. HBP is a multifunctional protein of inflammation that is responsible for a number of host response mechanisms to infection. HBP exhibits antimicrobial activity [6], induces recruitment of monocytes to the site of inflammation [7], and augments macrophage phagocytosis [8]. Besides its beneficial effects during the immune response, HBP is also a potent inducer of endothelial hyperpermeability [4]. For example, neutrophil degranulation and the associated release of HBP, can be triggered by the binding of fibrinogen-coated M protein of *streptococcus pyogenes* to β2 integrins, which causes a toxic shock syndrome characterized by plasma leakage and multi-organ failure [9].

Statins are inhibitors of hydroxyl-methylglutaryl coenzyme A reductase, the enzyme which catalyses the second reaction in the mevalonate-dependent biosynthesis pathway. Statins are being investigated as a potential therapy for ALI. Statins attenuate lung injury *in vivo* in mice which have received aerosolized lipopolysaccharide (LPS) [10] and attenuate vascular leak and inflammation in murine inflammatory lung injury [11]. In humans, oral intake of simvastatin for 4 days attenuates the systemic inflammatory response to intravenous LPS [12]. We have recently shown that simvastatin reduces pulmonary inflammation *in vivo* in healthy volunteers who have inhaled a low dose of LPS [13]. In this human model of ALI, simvastatin reduced LPS-induced BAL neutrophilia, tumour necrosis factor α, matrix metalloproteinases and C-reactive protein. In a recent randomized, double-blind trial of 60 patients with ALI (the HARP study), we showed that simvastatin induced modest improvement in non-pulmonary organ dysfunction [14]. The mechanisms by which simvastatin exerted its protective effect in the small single centre HARP study were unclear. We hypothesised that the levels of plasma HBP were elevated in patients with ALI and that the protective effects of simvastatin were mediated by reduced plasma HBP levels.

## Methods

### Study population

The ALI population used to carry out this study has been previously described in detail [14]. This was a single-centre, prospective, double-blind, randomized, placebo-controlled clinical trial in which patients with ALI were randomized to simvastatin 80 mg or placebo (1:1) for up to 14 days.

Baseline patient demographics, severity of illness scores were collected at baseline [14]. Briefly, 60 mechanically ventilated patients with ALI admitted to the regional Intensive Care Unit (ICU) in the Royal Victoria Hospital, Belfast, Northern Ireland, were recruited to the study. Five healthy subjects were recruited by advertising to undergo plasma sampling. Screening consisted of history, physical examination, routine blood investigation, electrocardiogram and spirometry. Healthy subjects included 4 males and one female. Mean baseline of FEV1 (L) and FVC (L) were 4.2(0.5) and 5.2(0.75), respectively. The study was approved by the local research ethics committee, and written informed consent was obtained from the legal representative of the patient or the healthy individuals. The research was carried out in compliance with the Helsinki Declaration.

### Preparation of plasma samples

Blood was collected in lithium heparin tubes (Becton-Dickinson, Plymouth, UK) and placed immediately on ice until processed. Plasma sampling for patients with ALI were performed at baseline, before study drug administration, and on day 3 and 7 as described [14]. Total cell count was determined using a haemocytometer.

### Measurement of HBP levels

Concentrations of HBP in plasma were determined using a validated ELISA as described [5].

### Statistical analysis

Data are expressed as median (interquartile range). Data were analyzed by unpaired t-test or Mann–Whitney U-test as appropriate. Data were analyzed using GraphPadPrism (version 4.02, GraphPad Software, San Diego, CA).

## Results

### HBP is increased in plasma at onset of ALI

**Figure 1:**
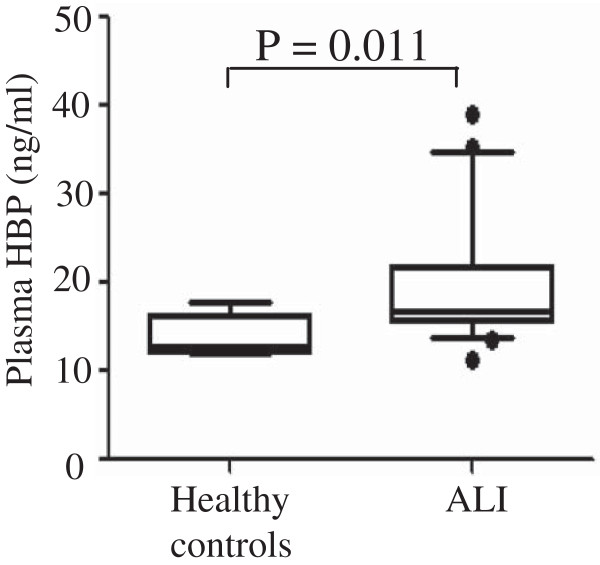
**Plasma levels of HBP in healthy individuals and patients with ALI.** Plasma samples were prepared from healthy controls and patients with ALI immediately after 48 h recruitment to ICU. Levels of plasma HBP were determined using a validated ELISA.

HBP concentration in plasma in healthy volunteers and patients with ALI were measured at enrollment. The median value of plasma HBP levels was 12.8 ng/ml in healthy subjects (n = 5) and 16.7 ng/ml in patients with ALI (n = 47). This corresponds to a 30% increase of HBP in patients with ALI (P = 0.011) (Figure 1).

### HBP levels and Intensive Care Unit mortality

**Figure 2:**
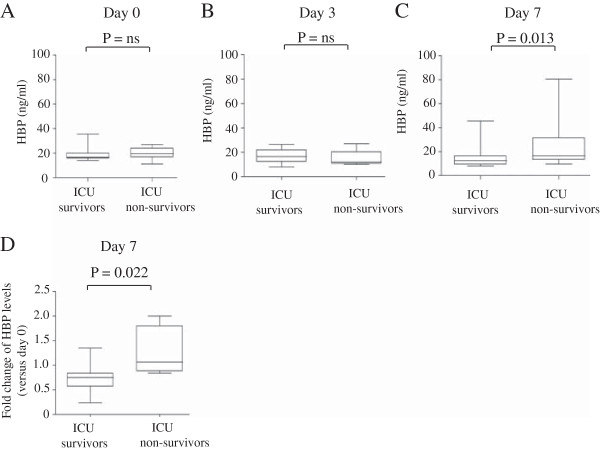
**ICU non**-**survivors have augmented plasma HBP level than ICU survivors.** Plasma HBP levels at day 0 **(A)**, day 3 **(B)**, or day 7 **(C)** in ICU survivors and ICU non-survivors are represented. **(D)** The fold change of HBP level at day 7 versus day 0 is represented in ICU survivors and ICU non-survivors.

We tested whether levels of HBP correlated with ICU mortality. To this end, we compared concentrations of plasma HBP in ICU survivors and ICU non-survivors at enrolment and after 3 and 7 days in ICU. At enrolment, and day 3, there was no significant difference in plasma HBP levels between ICU survivors (n = 21) and non-survivors (n = 9) (Figure 2A & B). However, at day 7 after enrolment, patients with ALI who did not survive had a significantly higher level of plasma HBP in comparison to ICU survivors (p = 0.013) (Figure 2C).

The fold change in plasma HBP levels at day 7 versus day 0 was significantly lower (p = 0.022) in ICU survivors (n = 21) than in ICU non-survivors (n = 9) (Figure 2D). Collectively, these results indicate that ICU survival is associated with decreased plasma HBP levels at day 7.

### Simvastatin reduces plasma HBP levels in patients with ALI

**Figure 3:**
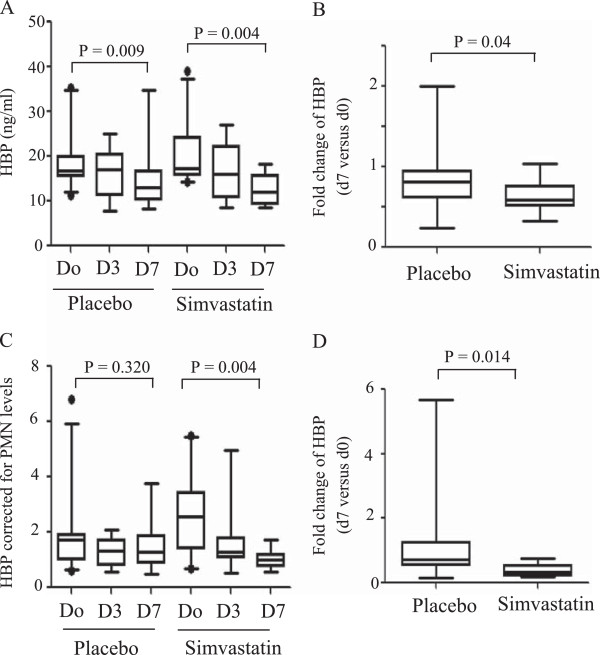
**Simvastatin decreases the level of plasma HBP in patients with ALI.(A)** Patients with ALI received either placebo or 80 mg simvastatin. Thereafter, plasma samples were collected at day 3 and day 7, and the levels of HBP were measured by ELISA. Baseline plasma HBP levels were measured before drug administration (day 0). **(B)** The fold change of HBP at day 7 versus day 0 was expressed for patients who have received placebo or 80 mg simvastatin. **(C)** The data in **(A)** were corrected to neutrophil counts. **(D)** The fold change of HBP corrected for neutrophil counts at day 7 versus day 0 was expressed for patients who have received placebo or 80 mg simvastatin.

We tested whether simvastatin could modify plasma HBP concentrations in patients with ALI. The level of plasma HBP decreased significantly at day 7 in both patients treated with placebo (n = 16, p = 0.009) or simvastatin (n = 12, p = 0.004) (Figure 3A). We found that the fold change of plasma HBP in patients with ALI treated with simvastatin was significantly lower than in placebo-treated patients (P = 0.04) (Figure 3B). This result indicates that a more pronounced reduction in plasma HBP levels is observed in patients with ALI treated with simvastatin.

We corrected the concentrations of plasma HBP for the number of neutrophils in blood. When the concentration of HBP was normalized for neutrophil counts, we found no significant decrease in HBP levels at days 3 and 7 after enrolment in placebo-treated patients (Figure 3C). In contrast, and similarly to above (Figures 3A & B), there was a significant decrease (p = 0.004) in HBP levels in patients treated with simvastatin at day 7 (Figure 3C). The fold change of HBP at day 7 versus day 0 in patients treated with simvastatin was significantly (p = 0.014) lower than in placebo-treated patients (Figure 3D).

## Discussion

Statins may have a beneficial role in ALI. We have recently shown that oral administration of 80 mg simvastatin to patients with ALI reduced pulmonary and systemic inflammation [14]. Simvastatin improved systemic organ failure with a trend to improvement in respiratory dysfunction [14]. Multi-organ dysfunction is caused, at least in part, by increased endothelial permeability and simvastatin has been shown to improve lung permeability in endotoxin-induced ALI in mice [15]. As HBP derived from neutrophils disrupts endothelial integrity causing vascular leakage and organ failure [4,9], we aimed to investigate the levels of plasma HBP in patients with ALI and if simvastatin modified those levels. As neutrophils play a central part in the pathogenesis of ALI [1], we hypothesised that HBP concentrations in plasma would be increased in patients with ALI. We found that plasma HBP levels in patients with ALI were significantly increased compared to healthy individuals. Other investigators found elevated levels of plasma HBP in patients with severe sepsis and septic shock [16,17] and HBP concentrations higher or equal to 15 ng/ml were found in 87% of critically ill patients with sepsis. Furthermore, when plasma HBP levels reached this threshold, there was a 4-fold increase of the mortality rate among patients with sepsis [16].

We next investigated whether plasma HBP level was associated with mortality. We found that plasma HBP levels at enrolment were not significantly different between patients with ALI surviving ICU admission or not. In contrast, other investigators found elevated HBP levels at admission in other diseases including sepsis, and this was associated with an increased mortality at 28 days [16]. We also observed that HBP levels decreased significantly at day 7 versus day 0 in ICU survivors but not in ICU non-survivors. Based on these findings, we propose that normalization of plasma HBP level over the time, rather than the concentration of HBP at enrolment, is associated with recovery. It is plausible that strategies to decrease plasma HBP levels may reduce endothelial leak and may be a potential therapeutic strategy to further investigate.

Simvastatin reduces pulmonary and systemic inflammation in patients with ALI [14] and in a human model of ALI induced by inhaled LPS [13]. Therefore, we investigated whether simvastatin reduced plasma HBP levels in patients with ALI. We found that patients treated with simvastatin had a greater fold reduction in plasma HBP levels at day 7 versus day 0 than those who received placebo. This relation persisted when corrected for neutrophil count suggesting this is not simply a reflect of reduced peripheral neutrophil counts but reduced neutrophil activation.

The relatively small sample size used in this study limits the interpretation of the data. Although we found that decreased plasma HBP levels was associated with survival and simvastatin decreased plasma levels of HBP in patients with ALI, simvastatin did not improve ALI survival [14]. It would be necessary to conduct a larger study in patients with ALI to confirm the association between the administration of simvastatin, reduced plasma levels of HBP, and survival which is ongoing [18].

Pro-inflammatory cytokines and chemokines, which characterise ALI, induce expression of β2 integrins (LFA-1 and Mac-1) on the membrane surface of neutrophils [19] and switch these adhesion receptors from a low to a high affinity ligand binding conformation [20]. This change in conformation allows the β2 integrins to bind endothelial ligands, which is a prerequisite for extravasation and activation of neutrophil inflammatory functions including degranulation (and release of HBP). Interestingly, statins have been shown to bind to an allosteric site within the β2 integrin LFA-1 which prevents LFA-1 interacting with endothelial ligands. This has been shown to impair migration of neutrophils in a murine peritonitis model [21]. Although statins do not bind Mac-1 [21], the dominant β2 integrin expressed in neutrophils, simvastatin has been shown to reduce expression of Mac-1 on the membrane surface of circulating neutrophils by reducing the level of pulmonary CXC chemokines [22]. Accordingly, potential mechanism for our finding is that simvastatin blocks β2 integrin-dependent release of HBP and other granule proteins.

## Conclusions

This study has shown that the concentration of HBP in plasma was increased in patients with ALI compared to healthy individuals. Furthermore, we found that at day 7 after onset of ALI, high plasma HBP levels was associated with mortality. Simvastatin was associated with greater reduction in plasma HBP. A greater reduction in plasma HBP may be one of the mechanism by which simvastatin exerts a potential protective effect.

## Abbreviations

ALI: Acute lung injury; ARDS: Acute respiratory distress syndrome; BAL: Bronchoalveolar lavage; HBP: Heparin-binding protein; LPS: Lipopolysaccharide.

## Competing interests

The authors do not have a financial relationship with a commercial entity that has an interest in the subject of this manuscript.

## Authors’ contributions

DFA designed the study, analyzed the data, and contributed to the writing of the manuscript. COK contributed to the analysis of the data and the writing of the manuscript. TRC and MS collected and analyzed the data, and contributed to the writing of the manuscript. HH contributed to the writing of the manuscript and provided technical expertise. KD initiated the study, analyzed the data, and wrote the manuscript. All authors read and approved the final manuscript.

## Pre-publication history

The pre-publication history for this paper can be accessed here:

http://www.biomedcentral.com/1471-2466/13/47/prepub
